# A comprehensive profiling of soluble immune checkpoints from the sera of patients with non‐small cell lung cancer

**DOI:** 10.1002/jcla.24224

**Published:** 2022-01-12

**Authors:** Ying Peng, Chen Zhang, Zhilian Rui, Weiming Tang, Yan Xu, Xiaoxin Tao, Qi Zhao, Xin Tong

**Affiliations:** ^1^ Department of Clinical Laboratory Liyang People’s Hospital Liyang China; ^2^ Center of Clinical Laboratory Medicine Zhongda Hospital Southeast University Nanjing China; ^3^ Department of Oncology Liyang People’s Hospital Liyang China; ^4^ Department of Respiratory and Critical Care Medicine Nanjing Drum Tower Hospital Nanjing University Medical School Nanjing China; ^5^ Department of Infectious Diseases Nanjing Drum Tower Hospital Nanjing University Medical School Nanjing China

**Keywords:** immunotherapy, non‐small cell lung cancer, soluble immune checkpoint

## Abstract

**Background:**

Immunotherapy was widely used for the treatment of non‐small cell lung cancer (NSCLC). However, whether inhibition of immune checkpoints individually or simultaneously could improve the therapeutic efficacy of NSCLC remains to be investigated. Here, we explored the aberrant levels of several checkpoints and evaluated their potential diagnostic values for NSCLC.

**Methods:**

Serum samples of 89 NSCLC patients and 57 healthy donors were collected from Nanjing Drum Tower Hospital between November 2019 and July 2020. Fourteen human immune checkpoints were quantified by Procarta‐Plex Human Immuno‐Oncology Checkpoint Panel.

**Results:**

The expression levels of sTIM‐3, sCD137, sCD27, sLAG‐3, sIDO, sPD‐L2, sCD152, sCD80, and sPD‐1 were all significantly increased in serum of NSCLC patients. Especially, sLAG‐3 was significantly elevated in serum of NSCLC patients at early‐stage (stages I and II), TIM‐3, CD137, and CD27 were significantly higher in the advanced NSCLC patients (stages III and IV) than in the early‐stage groups. Receiver operating characteristics (ROC) results showed that except for PD‐1, all the other immune checkpoint proteins had potential diagnostic values for NSCLC. sTIM‐3 had the highest diagnostic accuracy, followed by sLAG‐3. Combining sTIM‐3, sLAG‐3, and sCD137 could increase the accuracy to a higher level. Moreover, sCD27 was correlated with NSCLC cancer type, age, sex, and disease stage, while sCD137 was correlated with age and disease stage. sTIM‐3 and sIDO were correlated with stage and age, respectively.

**Conclusions:**

TIM‐3 and LAG‐3 were independent biomarkers for the early diagnosis of NSCLC. The combination of TIM‐3, LAG‐3, and CD137 could increase the diagnostic accuracy.

## Graphical abstract

- Serum samples of 89 NSCLC patients and 57 healthy donors were recruited to test soluble immune checkpoints.
- The expression levels of sTIM‐3, sCD137, sCD27, sLAG‐3, sIDO, sPD‐L2, sCD152, sCD80 and sPD‐1 were all significantly increased in serum of NSCLC patients.
- TIM3 and LAG3 were independent candidates for the early diagnosis of NSCLC. The combination of TIM‐3, LAG‐3, and CD137 could increase the diagnostic accuracy.
- Correlation analysis showed significant positive correlations between the levels of sTIM‐3, sCD137, and sCD27 and TNM stage of the patients, respectively.

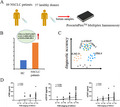

## 1 INTRODUCTION

Lung cancer is the most common malignancy and the leading cause of cancer‐related deaths worldwide.^1^ The main subtype of lung cancer is non‐small cell lung cancer (NSCLC), which accounts for more than 75% of the lung cancer cases.^2^ The main treatment options for NSCLC are surgery, radiotherapy, chemotherapy, and targeted therapy^3^; however, since most NSCLC patients were diagnosed at advanced stages, the 5‐year survival rate remained as low as 15.9%.^4^ Therefore, searching for new high‐sensitive biomarkers can be of great help for the diagnosis and treatment of NSCLC.

With the development of tumor immunology, tumor immunotherapy has become a new therapeutic approach in oncotherapy. The latest immunotherapeutic strategies aimed to target immune tolerance mechanisms by blocking immune checkpoints, thereby reversing functional suppression of the immune response, reactivating T cells, and promoting antitumor immunity.^5^ Immune checkpoints are expressed on immune cells that regulate the immune activation and played essential roles in preventing the onset of autoimmunity.^6^ Over the last decade, tumor immunotherapy that based on immune checkpoint blockade has attracted more and more attention.^7^ Immune checkpoint blockers targeting CTLA‐4 and PD‐1 have been widely used in clinics and achieved desired therapeutic efficacy.^8^

A number of studies have found that a variety of immune checkpoints played crucial roles in immune regulation by producing soluble molecules.^9^ For instance, the soluble form of the immune checkpoint molecule lymphocyte activation gene‐3 (LAG‐3), soluble LAG‐3 (sLAG‐3), was highly expressed in breast cancer and associated with poor prognosis.^10^ Meanwhile, the soluble form of the T‐cell immunoglobulin and mucin‐domain containing‐3 (TIM‐3), soluble TIM‐3 (sTIM‐3), may also play important roles in cancer development.^11^ A study showed that sTIM‐3 could promote tumor growth and inhibit T cell proliferation as well as IL‐2 and IFN‐γ secretion.^12^ Li et al. also illustrated that sTIM‐3 was prominently elevated in hepatocellular carcinoma (HCC) patients and may indicate a feasible prognosis HCC.^13^ These studies suggested that soluble molecules of immunosuppressive receptors may act in various ways to modulate the functions of membrane‐based immune checkpoint molecules. In‐depth studies on the soluble forms of immunosuppressive molecules and their biological activities were essential for improving immune checkpoint‐based tumor immunotherapy.

In this study, we examined 14 immune checkpoints in serum samples from NSCLC, evaluating their diagnostic values and their correlation with clinicopathological indicators. Our results may provide new theoretical basis for the diagnosis and treatment of NSCLC.

## 2 METHODS

### 2.1 Study population and data collection

Serum samples from 86 patients that pathologically confirmed as NSCLC were collected between November 2019 and July 2020 from Nanjing Drum Tower Hospital. All subjects had no history of any chemotherapy, radiotherapy, targeted therapy, or surgical resection. The medical records of the patients were reviewed to evaluate the clinicopathological features. Clinical data included the age, gender, smoking history, tumor histological type, and tumor‐node metastasis (TNM) stage of the patients. Nonsmokers were defined as patients with a smoking dose less than 100 cigarettes during their lifetime. Serum samples of 57 healthy donors were collected as the control group. The study was approved by the Human Ethics Committee of the Nanjing Drum Tower Hospital (Jiangsu, China), and written informed consent was obtained from each participant.

### 2.2 Detection of soluble immune checkpoint proteins in serum

The expression levels of 14 human immune checkpoint markers in serum samples were quantified using Procarta‐Plex Human Immuno‐Oncology Checkpoint Panel (Thermo Fisher, Waltham, MA). The assay was conducted according to protocols provided by the manufacturer using Luminex 200™ instrument and xPONENT^®^ software (Luminex Corp, Austin, TX). In brief, capture antibody‐conjugated beads were first added to the 96‐well plates, incubated for two minutes. The standards and serum samples were then added to the plates containing the universal assay buffer. Next, the plates were incubated at room temperature for 30 min followed by overnight incubation at 4 °C on a shaker (500 rpm). Biotinylated detection antibodies were then added, and the plates were incubated at room temperature for 30 min. After incubation, the plates were washed, treated by streptavidin‐phycoerythrin and incubated at room temperature for 30 min. The plates were then washed, and a reading buffer was added to the wells. Finally, a Luminex 200™ instrument was used to read the plates.

### 2.3 Detection of CEA, NSE, and CYFRA21‐1 in serum samples

A total of 5 mL of fasting blood was collected from each participant early in the morning; then the serum samples were separated by centrifugation. The levels of carcinoembryonic antigen (CEA), neuron‐specific enolase (NSE), and cytokeratin 19 fragment antigen 21–1 (CYFRA21‐1) were measured by Roche Cobas e601 automatic electrochemiluminescence meter using commercialized reagents (Jianglaibio, Shanghai, China).

### 2.4 Statistical analysis

All statistics were performed using SPSS 19.0 software (IBM) and GraphPad Prism 7.0 (GraphPad Inc.). Since the soluble immune checkpoint proteins did not conform to the normal distribution, they expressed as the median (M) and interquartile range (IQR). The Mann–Whitney *U* test was used to compare groups, and spearman correlation analysis was performed to test the correlation. In addition, the receiver operating characteristic (ROC) curve was used to select the cut‐off values to calculate the sensitivity, specificity, and accuracy. For all analysis, a two‐sided *p* < 0.05 was considered significant statistically.

## 3 RESULTS

### 3.1 Analysis of clinical data of patients

Eighty‐six NSCLC patients were enrolled in the study; the clinical and pathological features were summarized (Table 1). Adenocarcinoma and squamous carcinoma subtypes accounted for 53.49% and 46.51%, respectively. According to the 8th edition of TNM staging of the Union for International Cancer Control (UICC), 5 cases were classified as stage I, 19 as stage II, 21 as stage III, and 41 as stage IV. Moreover, the mean age of NSCLC patients and healthy controls was 66 versus 65. There were no statistical differences between gender, age, and smoking history between the two groups.

**TABLE 1:** Clinical feature of NSCLC patients and healthy controls

Characteristics	NSCLC patients (*n *= 86)	Healthy controls (*n *= 57)
Age		
≤65	34 (39.53)	22 (38.60)
>65	52 (60.47)	35 (61.40)
Gender		
Male	66 (76.74)	42 (73.68)
Female	20 (23.26)	15 (26.32)
Smoking		
Yes	46 (53.49)	31 (54.39)
No	40 (46.51)	26 (45.61)
Histology		
Adenocarcinoma	46 (53.49)	NA
Sq. cell carcinoma	40 (46.51)	NA
TNM stage		NA
Stage I	5 (5.81)	NA
Stage II	19 (22.09)	NA
Stage III	21 (24.42)	NA
Stage IV	41 (47.67)	NA

### 3.2 Determination of serum levels of the immune checkpoint proteins in NSCLC patients

Results of Luminex tests determined the expression profile of soluble immune checkpoint proteins. As shown in Table 2, compared with the control group, 9 markers were overexpressed in NSCLC patients, including soluble TIM‐3, CD137, CD27, LAG‐3, IDO, PD‐L2, CD152, CD80, and PD‐1 (0.92, 0.27, 74.15, 2.68, 6.11, 760.00, 1.65, 6.32, and 7.29 pg/ml, respectively). Soluble PD‐L1, HVEM, GITR, BTLA, and CD28 were rarely expressed in serum samples.

**TABLE 2:** Expressions of soluble immune checkpoint proteins in NSCLC group and normal control group [M (QR), pg/ml]

Soluble immune checkpoint proteins	NSCLC group (*n *= 86)	Control group (*n *= 57)	*Z*	*p*
sTIM−3	0.92 (88.21)	0.38 (0.37)	−5.300	<0.001
sCD137	0.27 (6.54)	0.06 (0.13)	−3.845	<0.001
sCD27	74.15 (97.60)	51.61 (43.30)	−3.581	<0.001
sLAG−3	2.68 (6.98)	0.94 (0.56)	−5.529	<0.001
sIDO	6.11 (12.71)	3.36 (4.14)	−2.623	0.009
sPD‐L2	760.00 (329.78)	655.65 (247.83)	−2.268	0.023
sCD152	1.65 (0.75)	0.90 (1.65)	−2.133	0.033
sCD80	6.32 (2.10)	5.91 (2.21)	−1.993	0.046
sPD−1	7.29 (8.70)	5.34 (4.20)	−1.025	0.049

### 3.3 Association between the expressions of immune checkpoint proteins and clinicopathological characteristics of the patients

We investigated the relationship between the expression levels of sTIM‐3, sCD137, sCD27, sLAG‐3, sIDO, Spd‐L2, sCD152, sCD80, sPD‐1, and the clinicopathological features (age, gender, smoking history, histological subtype, and TNM stage) of NSCLC patients. As shown in Table S1, sCD137 was strongly correlated with advanced age (>65) (*r* = 0.225, *p *= 0.038) and advanced TNM stage (stages III and IV) (*r* = 0.230, *p* = 0.033). Increased sCD27 was associated with advanced age (>65) (r = 0.239, *p* = 0.027), male sex (*r* = −0.235, *p *= 0.030), adenocarcinoma subtype (*r* = −0.324, *p *= 0.002), and advanced TNM stage (stages III and IV) (*r* = 0.273, *p *= 0.011). Furthermore, the levels of sTIM‐3 and sIDO were positively related with the TNM stage (stages III and IV) (*r* = 0.305, *p* = 0.004) and age of the patients (*r *= 0.344, *p *= 0.001), respectively.

As shown in Table 3, the expression levels of sTIM‐3, sCD137, sLAG‐3, and sIDO in both lung adenocarcinoma and lung squamous cell carcinoma patients were significantly higher than those of healthy controls. However, sPD‐L2 and sPD‐1 were only overexpressed in lung adenocarcinoma patients, and the serum levels of CD27 and CD152 were markedly elevated in patients with lung squamous cell carcinoma, but not lung adenocarcinoma patients. The squamous cell carcinoma group had higher serum level of sCD27 than the adenocarcinoma group (*p *= 0.003). In contrast, the other eight markers showed no significant differences between the two groups.

**TABLE 3:** Expressions of soluble immune checkpoint proteins in ADC group and SQCC group [M (QR), pg/ml]

Soluble immune checkpoint proteins	ADC group (*n *= 46)	SQCC group (*n *= 40)	Control group (*n *= 57)	*p* value Control vs. ADC	*P* Value Control vs. SQCC	*p* Value ADC vs. SQCC
sTIM−3	0.84 (44.95)	1.18 (105.65)	0.38 (0.37)	<0.001	<0.001	0.112
sCD137	0.29 (6.50)	0.15 (8.05)	0.06 (0.13)	<0.001	0.005	0.538
sCD27	59.91 (85.08)	108.48 (117.24)	51.61 (43.30)	0.160	<0.001	0.003
sLAG−3	2.68 (7.72)	2.68 (6.64)	0.94 (0.56)	<0.001	<0.001	0.839
sIDO	5.60 (8.61)	7.06 (13.79)	3.36 (4.14)	0.029	0.024	0.700
sPD‐L2	782.16 (320.59)	728.95 (358.74)	655.65 (247.83)	0.022	0.131	0.668
sCD152	0.90 (0.75)	1.65 (1.24)	0.90 (1.65)	0.199	0.018	0.225
sCD80	6.45 (1.70)	6.32 (2.65)	5.91 (2.21)	0.102	0.083	0.849
sPD−1	8.43 (6.92)	5.97 (10.44)	5.34 (4.20)	0.019	0.597	0.112

**FIGURE 1:**
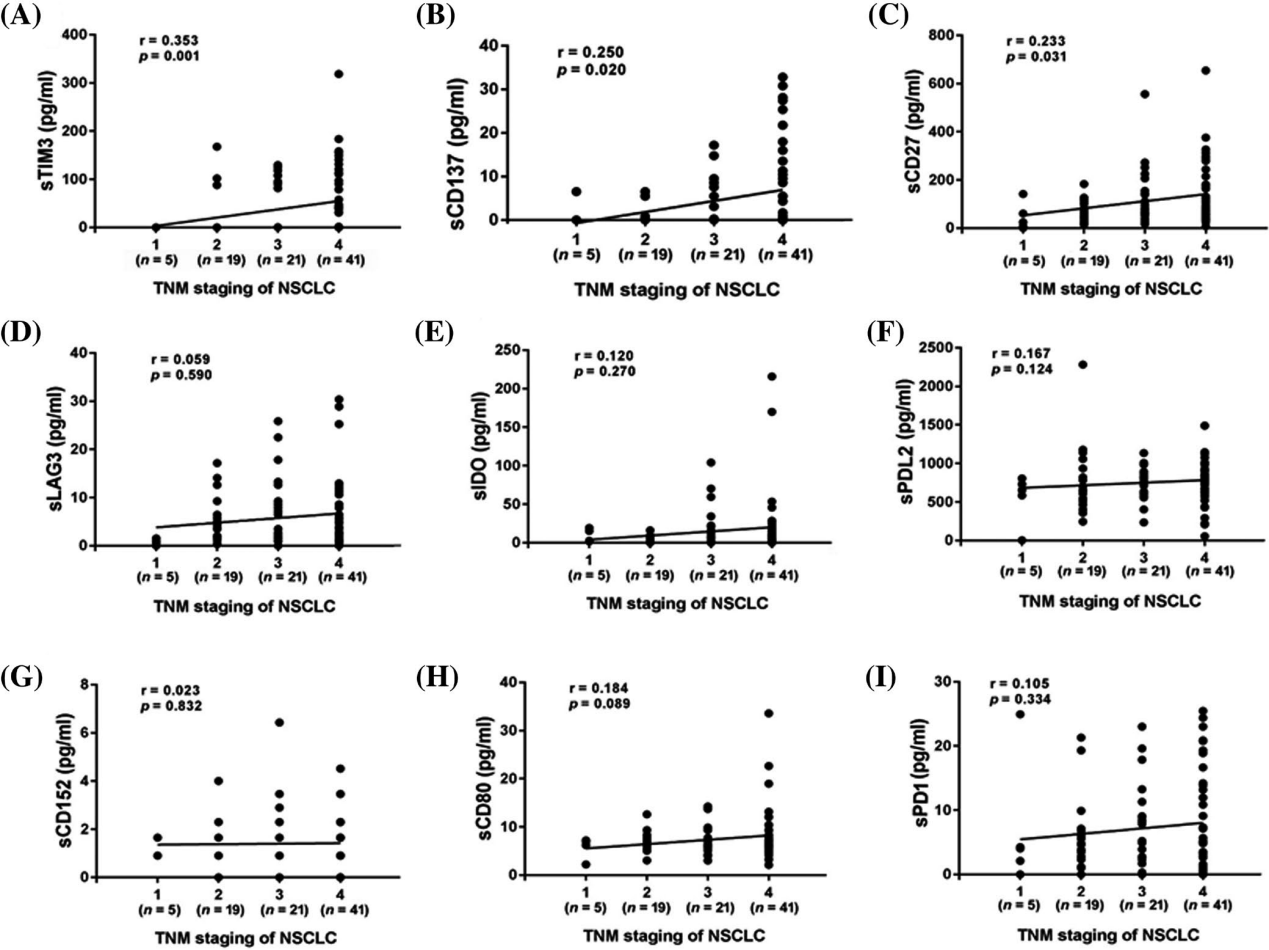
Correlations between serum protein markers and TNM staging of NSCLC. (A‐I) Correlations between TNM staging and, (A) sTIM3, (B) sCD137, (C) sCD27, (D) sLAG3, (E) sIDO, (F) sPDL2, (G) sCD152, (H) sCD80, (I) sPD1. Spearman’s rank correlation test was used. *p* value<0.05 was considered statistically significant

Moreover, compared with the controls, the expression levels of the nine markers were all significantly increased in serum of NSCLC patients at advanced stage. Compared with the healthy group, sLAG‐3 was dramatically increased in patients with early‐stage lung cancer (stages I and II), while the other markers showed no significant difference. In addition, sTIM‐3, sCD137, and sCD27 were significantly higher in the advanced lung cancer group than those in the early lung cancer group (Table 4). Correlation analysis also showed significant positive correlations between the levels of sTIM‐3, sCD137, and sCD27, and TNM stage of the patients, respectively (Figure 1).

**TABLE 4:** Comparison of the expressions of soluble immune checkpoint proteins among the three groups (healthy subjects, early and advanced NSCLC patients) [M (QR), pg/ml]

Soluble immune checkpoint proteins	Early stage (I/II)	Advanced stage (III/IV)	Control	*p* value control vs. early	*p* value control vs. advanced	*P* value early vs. advanced
sTIM−3	0.58 (1.16)	1.28 (98.93)	0.38 (0.37)	0.096	<0.001	0.005
sCD137	0.10 (0.88)	0.30 (9.46)	0.06 (0.13)	0.176	<0.001	0.034
sCD27	61.84 (83.51)	84.69 (125.91)	51.61 (43.30)	0.495	<0.001	0.012
sLAG−3	2.75 (4.28)	2.68 (7.71)	0.94 (0.56)	<0.001	<0.001	0.467
sIDO	5.44 (4.88)	6.80 (14.98)	3.36 (4.14)	0.423	0.003	0.121
sPD‐L2	653.93 (424.35)	782.16 (292.77)	655.65 (247.83)	0.748	0.006	0.220
sCD152	0.90 (0.75)	1.65 (0.91)	0.90 (1.65)	0.212	0.035	0.591
sCD80	6.19 (1.89)	6.70 (2.62)	5.91 (2.21)	0.626	0.020	0.131
sPD−1	5.90 (4.39)	7.83 (10.10)	5.34 (4.20)	0.672	0.030	0.265

### 3.4 The correlation analysis between the soluble immune checkpoint proteins

As shown in Table 5, there were significant positive correlations (*p *< 0.05) between any two soluble immune checkpoint proteins except for sCD152 and sCD80. sCD152 was correlated with sCD27 only (*r* = 0.259, *p *= 0.016). There was no significant correlation between sCD80 and sCD137.

**TABLE 5:** Correlation analysis of serum protein markers in NSCLC group [*r* (*p*)]

NSCLC group	NSCLC group								
	sTIM−3	sCD137	sCD27	sLAG−3	sIDO	sPD‐L2	sCD152	sCD80	sPD−1
sTIM−3	1.000	0.383 (<0.001)^**^	0.402 (<0.001)**	0.324 (0.002)**	0.301 (0.005)**	0.408 (<0.001)**	0.117 (0.285)	0.322 (0.003)**	0.360 (0.001)**
sCD137	0.383 (<0.001)**	1.000	0.376 (<0.001)**	0.419 (<0.001)**	0.418 (<0.001)**	0.352 (0.001)**	0.011 (0.921)	0.194 (0.074)	0.460 (<0.001)**
sCD27	0.402 (<0.001)**	0.376 (<0.001)**	1.000	0.473 (<0.001)**	0.397 (<0.001)**	0.349 (0.001)**	0.259 (0.016)*	0.314 (0.003)**	0.344 (0.001)**
sLAG−3	0.324 (0.002)**	0.419 (<0.001)**	0.473 (<0.001)**	1.000	0.261 (0.015)*	0.534 (<0.001)**	0.096 (0.379)	0.259 (0.016)*	0.399 (<0.001)**
sIDO	0.301 (0.005)**	0.418 (<0.001)**	0.397 (<0.001)**	0.261 (0.015)*	1.000	0.354 (0.001)**	0.116 (0.288)	0.409 (<0.001)**	0.358 (0.001)**
sPD‐L2	0.408 (<0.001)**	0.352 (0.001)**	0.349 (0.001)**	0.534 (<0.001)**	0.354 (0.001)**	1.000	0.088 (0.421)	0.246 (0.023)*	0.365 (0.001)**
sCD152	0.117 (0.285)	0.011 (0.921)	0.259 (0.016)*	0.096 (0.379)	0.116 (0.288)	0.088 (0.421)	1.000	0.193 (0.076)	0.178 (0.102)
sCD80	0.322 (0.003)**	0.194 (0.074)	0.314 (0.003)**	0.259 (0.016)*	0.409 (<0.001)**	0.246 (0.023)*	0.193 (0.076)	1.000	0.354 (0.001)**
sPD−1	0.360 (0.001)**	0.460 (<0.001)**	0.344 (0.001)**	0.399 (<0.001)**	0.358 (0.001)**	0.365 (0.001)**	0.178 (0.102)	0.354 (0.001)**	1.000

*When the confidence level (both sides) is 0.05, the correlation is significant.

**When the confidence level (both sides) is 0.01, the correlation is significant.

### 3.5 Diagnostic value analysis of soluble immune checkpoint proteins for NSCLC

**FIGURE 2:**
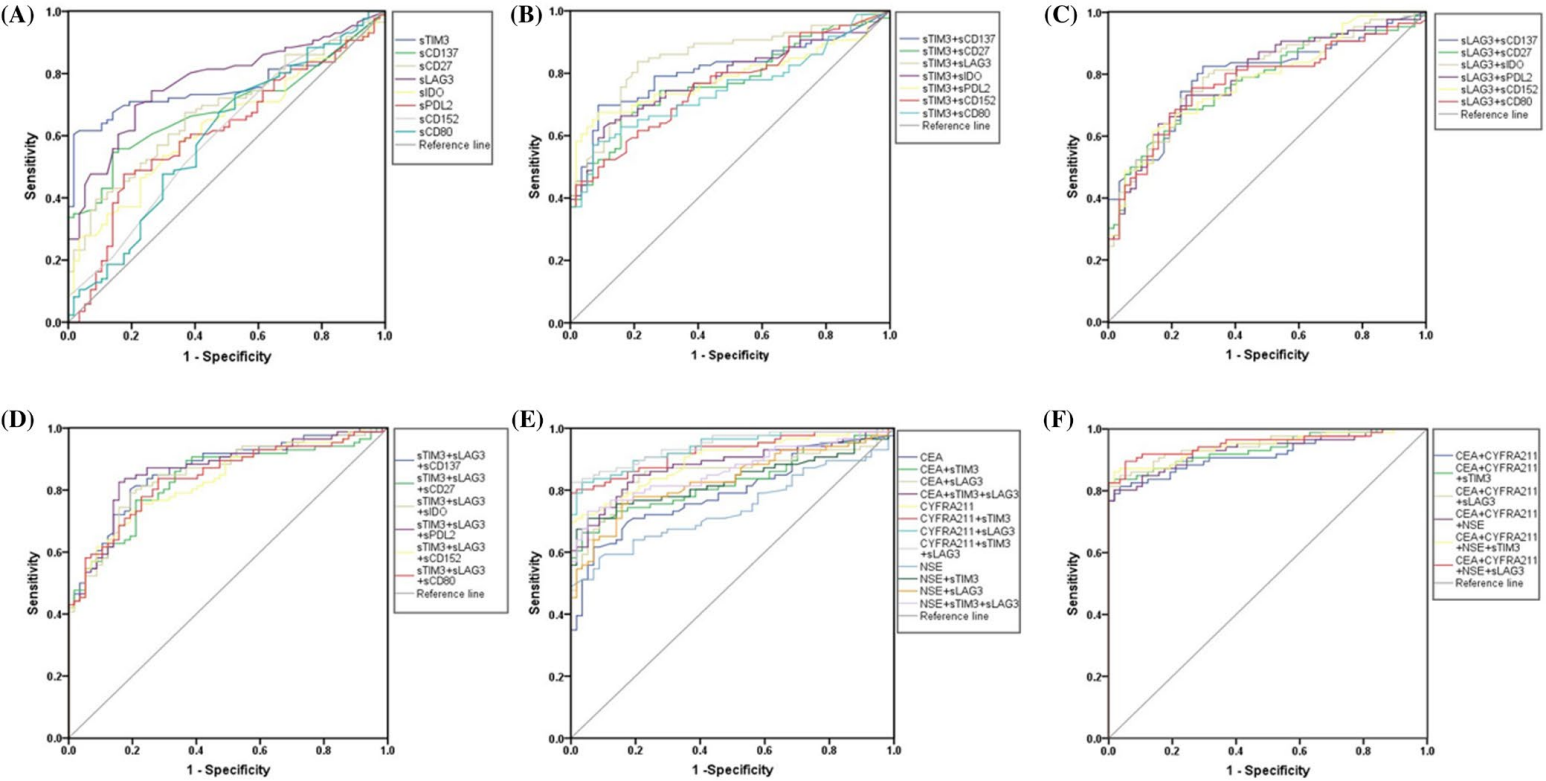
The ROC curve of soluble immune checkpoint proteins on NSCLC

The potential diagnostic value of single or combined soluble immune checkpoint proteins for NSCLC was evaluated (Table 6, Table S2, Table S3, and Figure 2). As shown in Table 6 and Figure 2, except for sPD‐1, all the other immune checkpoint proteins had potential diagnostic value for NSCLC. sTIM‐3 has the highest level of accuracy (75.53%), and the area under the curve (AUC) was 0.761(95% CI, 0.682–0.841, *p *< 0.001), with sensitivity and specificity of 60.47% and 98.25%, respectively. The accuracy of sLAG‐3 was 73.43%, and AUC was 0.774 (95% CI, 0.6972–0.8497, *p*<0.001; sensitivity, 69.77%; specificity, 78.95%). Results in Table S2 illustrated that combined detection of sTIM‐3, sLAG‐3, and sCD137 increased the accuracy to a high level (93.01%) and the AUC of 0.864 (95% CI, 0.805–0.923, *p *< 0.001; sensitivity, 89.53%; specificity, 80.00%). Regular lung cancer tumor markers including CEA, NSE, and CYFRA21‐1 were tested alone and combined with soluble immune checkpoint proteins, and their diagnostic values for NSCLC were shown in Tables S2 and S3. The AUCs of sTIM‐3 (AUC = 0.761) and sLAG‐3 (AUC = 0.774) combination were higher than NSE (AUC = 0.741), close to CEA (AUC =0.794) and lower than CYFRA21‐1 (AUC = 0.897). When regular tumor markers were combined with immune checkpoint proteins, the AUCs were all increased, but the differences were not significant.

**TABLE 6:** ROC curve analysis of single soluble immune checkpoint proteins

Index	Area under curve	OR	95%CI	*p* value	Sensitivity (%)	Specificity (%)	Accuracy (%)
sTIM−3	0.761	0.041	0.682–0.841	<0.001	60.47	98.25	75.53
sCD137	0.688	0.044	0.603–0.774	<0.001	54.65	85.96	67.13
sCD27	0.677	0.044	0.591–0.764	<0.001	39.53	91.23	60.14
sLAG−3	0.774	0.039	0.697–0.850	<0.001	69.77	78.95	73.43
sIDO	0.630	0.046	0.539–0.720	0.009	46.51	77.19	58.74
sPD‐L2	0.612	0.048	0.518–0.706	0.023	47.67	82.46	61.54
sCD152	0.602	0.048	0.508–0.697	0.039	51.16	63.16	55.94
sCD80	0.599	0.049	0.503–0.695	0.046	73.26	47.37	62.94
sPD−1	0.542	0.048	0.448–0.636	0.396	39.53	78.95	55.24

## 4 DISCUSSION

ProcartaPlex^TM^ immunoassays incorporate magnetic microsphere technology licensed from Luminex^TM^ to enable the simultaneous detection and quantitation of multiple protein targets in diverse matrices. In cases where commercially available assays are not available, the Luminex platform is superior to many other multiplex immunoassay platforms because custom assays can be developed with relative ease.^14^ The platform allows the simultaneous detection from a single sample of up to 80 protein targets on the Luminex^TM^ 200^TM^ and FLEXMAP 3D^TM^ platform and 50 protein targets on the MAGPIX^TM^ platform.

For the first time, we performed simultaneous quantification of 14 soluble immune checkpoint proteins in patients with NSCLC using multiplexed immunobead assay. These proteins included soluble forms of both stimulatory and inhibitory factors that regulate the activation and proliferation of T cells involved in the cancer‐immunity cycle. Using this extensive profiling, we identified significant changes not only for commonly studied inhibitory factors such as sPD‐1, sTIM‐3 or s LAG‐3 but also for less well‐studied checkpoint proteins.

In our results, we found an increased level of sTIM‐3 in serum samples from NSCLC patients. Meanwhile, sTIM‐3 expression was correlated with an advanced stage of TNM (stages III and IV) and age. Also, the expression of sTIM‐3 was higher in both lung adenocarcinoma and lung squamous cell lung carcinoma than those in the healthy subjects. Moreover, the serum level of sTIM‐3 was also markedly increased in the advanced stage group than in the early‐stage group. Furthermore, sTIM‐3 has an AUC of 0.761 (95% CI = 0.682–0.841), a sensitivity of 60.47%, a specificity of 98.25%, and an accuracy of 75.53%. Our results suggested that sTIM‐3 may function as an independent diagnostic indicator of NSCLC. Additional studies have reported the presence of sTIM‐3 abnormalities in different cancers. For instance, a study reported that sTIM‐3 was significantly elevated in the serum of patients with HCC.^13^, ^15^ Besides, sTIM‐3 was remarkably increased in osteosarcoma patients, strongly correlated with larger tumor size, late stages, distant metastases, and unfavorable prognosis.^16^ Previous studies have suggested that patients with higher sTIM‐3 expression presented a lower survival rate in NSCLC.^17^ In addition, consistent with our results, TIM‐3 expression characterized regulatory T cells in tumor tissues was associated with lung cancer progression.^18^

We also investigated the levels of other soluble immune checkpoint proteins including the stimulatory factor CD27 and inhibitory factors such as CD152 (also called CTLA‐4) and LAG‐3. As a member of the TNFR superfamily, CD27 can regulate T‐cell immunity, B‐cell activation, and immunoglobulin synthesis by binding to CD70.^19^, ^20^ Moreover, high serum soluble CD27 level correlated with poor performance status and reduced survival in patients with advanced lung cancer.^21^ It has been shown that under normal conditions, CD152 was localized intracellularly, and its expression on the cell surface was low.^22^ Since CD152 can inhibit the tyrosine phosphorylation of T cells after activation by binding to T‐cell receptor (TCR) chains, exerting its negative immune regulatory effect.^23^ The soluble monomeric form of LAG‐3 was often detected in patients with inflammatory diseases, and its expression level increased when patients were effectively treated.^24^, ^25^ In addition, previous studies suggested that the level of sLAG‐3 in the serum of patients with early‐stage NSCLC was significantly higher than that of patients with advanced‐stage NSCLC.^19^, ^26^ In our study, sCD27, sCD152, and sLAG‐3 were highly expressed in the sera of NSCLC patients. The expression of sLAG‐3 was higher in both ADC and SQCC than in the healthy group, while sCD152 was only highly expressed in the ADC group and sCD27 was only abundantly expressed in the SQCC group. The expression of sLAG‐3 was significantly higher in early‐stage NSCLC patients compared to the healthy group. In particular, sCD27 was expressed at significantly higher levels in the serum of patients with SQCC than in ADC. Moreover, the expression of sCD27 was also significantly higher in the advanced NSCLC group than in the early‐stage group. The AUCs of sCD27, sCD152, and sLAG‐3 in NSCLC were 0.677, 0.602, and 0.774.

PD‐1 was a novel costimulatory molecule, and binding to its ligand PD‐L1 may inhibit the effect of T cells.^27^ sPD‐1 may be involved in the activation of T cells and the immune response process by blocking the PD‐1/PD‐L1 negative signaling pathway.^28^ Studies have shown that PD‐L2 interaction with PD‐1 on activated T cells significantly inhibited the biological functions of effector T cells and the production of IL‐2.^29^ Here, we demonstrated that sPD‐1 and sPD‐L2 were only highly expressed in the ADC group. The diagnostic analysis revealed the AUCs of sPD‐1 and sPD‐L2 with 0.542 and 0.612, respectively. Currently, numerous studies have reported that sPD‐1 and sPD‐L1 might play important roles in the initiation, promotion, and progression of NSCLC. However, it was not detected in our assay system due to low sPD‐L1 serum levels.

Among soluble stimulatory factors, sCD137, sIDO, and sCD80L levels were also elevated in the serum of NSCLC. The expression of sCD137 was significantly higher in both lung adenocarcinoma and lung squamous cell lung carcinoma groups; moreover, the expression of sCD137 was also significantly higher in the advanced NSCLC group than that in the early‐stage group, while the AUC of sCD137 in NSCLC was 0.688 (95% CI =0.603–0.774; specificity =85.96%, sensitivity =54.65%, accuracy =67.13%). From the data in our study, sIDO was highly expressed in NSCLC and correlated with stage and age. The diagnostic analysis displayed an AUC of sIDO in NSCLC of 0.630 (95% CI =0.539–0.720). IDO was a rate‐limiting enzyme that catalyzed tryptophan catabolism and played an important role in immune tolerance in T cells and tumor immune escape.^22^, ^30^

More importantly, independent assays for soluble immune checkpoints and common lung cancer tumor markers CEA, NSE, and CYFRA21‐1 showed all of the immune checkpoints except sPD‐1 had diagnostic potentials in the NSCLC detection. sTIM‐3 had the highest diagnostic accuracy (75.53%), with the second‐highest accuracy being sLAG‐3 (73.4%), both lower than CEA (79.4%), CYFRA21‐1 (89.7%), or NSE (74.1%). The results of the combined detection assays showed that the combination of sTIM‐3, sLAG‐3, and sCD137 increased the diagnostic accuracy to a high level (93.0%) with the AUC to 0.864.

This study has some limitations. First, the number of our samples is relatively small, and some immune checkpoint proteins might be statistically significant if we expand the number of samples. Second, the sensitivity of Luminex is not as high as ELISA, which may cause some soluble immune checkpoint below the limit of detection.

In conclusion, TIM‐3 and LAG‐3 could be accurate, sensitive, and specific independent biomarkers in NSCLC. Combining testing of TIM‐3, LAG‐3, and CD137 could enhance the early diagnostic accuracy of NSCLC.

## CONFLICT OF INTEREST

The authors have declared that no conflict of interest exists.

## AUTHOR CONTRIBUTIONS

XT and YP designed the study. ZR and QZ recruited the patients. CZ and WT processed the blood samples, XT and YP performed the antibody assay. YX and ZR analyzed and interpreted the data. YP, XT and CZ wrote the manuscript. All the authors revised the manuscript.

## Supporting information

Table S1‐S3

## Data Availability

The data that support the findings of this study are available from the corresponding author upon reasonable request.
