# Supplementary material for: A comprehensive profiling of soluble immune checkpoints from the sera of patients with non‐small cell lung cancer
Source: J Clin Lab Anal. 2022 Jan 12;36(2):e24224. doi: 10.1002/jcla.24224 (PMC8841185; doi:10.1002/jcla.24224)
Supplement: Supplementary file 1 — Table S1‐S3 [file JCLA-36-e24224-s001.docx]

|  |  | histological subtype(1,2) | gender(1,2) | age(1,2) | smoking history (0,1) | TNM stage  (1-4) | TNM stage  (early- advanced) |
| --- | --- | --- | --- | --- | --- | --- | --- |
| TIM3 | r | -.172 | -.147 | .092 | .117 | .353 | .305 |
|  | p | .113 | .178 | .398 | .284 | .000 | .004 |
| CD137 | r | .067 | -.078 | .225 | .033 | .250 | .230 |
|  | p | .541 | .474 | .038 | .762 | .020 | .033 |
| CD27 | r | -.324 | -.235 | .239 | .020 | .233 | .273 |
|  | p | .002 | .030 | .027 | .854 | .031 | .011 |
| IDO | r | -.042 | -.177 | .344 | -.067 | .120 | .168 |
|  | p | .702 | .103 | .001 | .539 | .270 | .122 |
| PD-L2 | r | .046 | -.014 | .080 | -.176 | .167 | .133 |
|  | p | .671 | .899 | .467 | .105 | .124 | .222 |
| LAG3 | r | -.022 | -.040 | .046 | -.126 | .059 | .079 |
|  | p | .840 | .715 | .674 | .246 | .590 | .470 |
| PD-1 | r | .171 | -.094 | .156 | -.026 | .105 | .118 |
|  | p | .114 | .391 | .151 | .813 | .334 | .279 |
| CD152 | r | -.132 | -.170 | .096 | .096 | .023 | .058 |
|  | p | .227 | .118 | .381 | .378 | .832 | .594 |
| CD80 | r | -.021 | -.089 | .064 | -.129 | .184 | .164 |
|  | p | .850 | .416 | .560 | .236 | .089 | .132 |

Table S1 Correlation between the expression levels of soluble immune checkpoints and the clinicopathological features of NSCLC patients

| Table S2. ROC curve analysis of combined detection of serum protein markers. | | | | | | | |
| --- | --- | --- | --- | --- | --- | --- | --- |
| Index | Area under curve | OR | 95%CI | *P* Value | Sensitivity(%) | Specificity(%) | Accuracy(%) |
| sTIM3 | 0.761 | 0.041 | 0.682-0.841 | <0.001 | 60.47 | 98.25 | 75.53 |
| sTIM3+sCD137 | 0.810 | 0.036 | 0.739-0.881 | <0.001 | 74.42 | 86.15 | 83.92 |
| sTIM3+sCD27 | 0.772 | 0.039 | 0.697-0.848 | <0.001 | 68.60 | 89.47 | 76.92 |
| sTIM3+sLAG3 | 0.849 | 0.032 | 0.785-0.912 | <0.001 | 83.72 | 77.19 | 81.12 |
| sTIM3+sIDO | 0.784 | 0.038 | 0.710-0.858 | <0.001 | 73.26 | 75.44 | 74.13 |
| sTIM3+sPDL2 | 0.786 | 0.038 | 0.711-0.861 | <0.001 | 72.09 | 80.70 | 75.52 |
| sTIM3+sCD152 | 0.761 | 0.039 | 0.685-0.838 | <0.001 | 80.23 | 61.40 | 72.73 |
| sTIM3+sCD80 | 0.749 | 0.040 | 0.670-0.827 | <0.001 | 88.37 | 47.37 | 72.03 |
| sLAG3 | 0.774 | 0.039 | 0.697-0.850 | <0.001 | 69.77 | 78.95 | 73.43 |
| sLAG3+sCD137 | 0.795 | 0.037 | 0.722-0.868 | <0.001 | 82.56 | 81.16 | 88.81 |
| sLAG3+sCD27 | 0.778 | 0.038 | 0.703-0.852 | <0.001 | 70.93 | 70.18 | 70.63 |
| sLAG3+sIDO | 0.799 | 0.037 | 0.727-0.871 | <0.001 | 80.23 | 70.18 | 76.22 |
| sLAG3+sPDL2 | 0.792 | 0.037 | 0.718-0.865 | <0.001 | 74.42 | 70.18 | 72.73 |
| sLAG3+sCD152 | 0.780 | 0.038 | 0.706-0.854 | <0.001 | 81.40 | 50.88 | 69.23 |
| sLAG3+sCD80 | 0.772 | 0.039 | 0.696-0.849 | <0.001 | 89.53 | 42.11 | 70.63 |
| sTIM3+sLAG3 | 0.849 | 0.032 | 0.785-0.912 | <0.001 | 83.72 | 77.19 | 81.12 |
| sTIM3+sLAG3+sCD137 | 0.864 | 0.030 | 0.805-0.923 | <0.001 | 89.53 | 80.00 | 93.01 |
| sTIM3+sLAG3+sCD27 | 0.834 | 0.034 | 0.768-0.900 | <0.001 | 84.88 | 68.42 | 78.32 |
| sTIM3+sLAG3+sIDO | 0.850 | 0.032 | 0.788-0.912 | <0.001 | 88.37 | 68.42 | 80.42 |
| sTIM3+sLAG3+sPDL2 | 0.864 | 0.031 | 0.804-0.924 | <0.001 | 84.88 | 68.42 | 78.32 |
| sTIM3+sLAG3+sCD152 | 0.830 | 0.033 | 0.765-0.895 | <0.001 | 89.53 | 49.12 | 73.43 |
| sTIM3+sLAG3+sCD80 | 0.839 | 0.033 | 0.775-0.903 | <0.001 | 95.35 | 42.11 | 74.13 |

| Table S3. ROC curve analysis of serum protein markers combined with common tumor markers. | | | | | | | |
| --- | --- | --- | --- | --- | --- | --- | --- |
| Index | Area under curve | OR | 95%CI | *P* Value | Sensitivity(%) | Specificity(%) | Accuracy(%) |
| CEA | 0.794 | 0.037 | 0.722-0.866 | <0.001 | 61.63 | 92.98 | 74.13 |
| CEA+sTIM3 | 0.829 | 0.034 | 0.762-0.895 | <0.001 | 79.07 | 91.23 | 83.92 |
| CEA+sLAG3 | 0.856 | 0.032 | 0.792-0.919 | <0.001 | 84.88 | 75.44 | 81.12 |
| CEA+sTIM3+sLAG3 | 0.880 | 0.029 | 0.824-0.936 | <0.001 | 93.02 | 73.68 | 85.31 |
| CYFRA211 | 0.897 | 0.026 | 0.847-0.947 | <0.001 | 69.77 | 100.00 | 81.82 |
| CYFRA211+sTIM3 | 0.925 | 0.022 | 0.882-0.968 | <0.001 | 84.88 | 98.25 | 90.21 |
| CYFRA211+sLAG3 | 0.937 | 0.019 | 0.899-0.975 | <0.001 | 90.70 | 78.95 | 86.01 |
| CYFRA211+sTIM3+sLAG3 | 0.945 | 0.018 | 0.909-0.980 | <0.001 | 94.19 | 77.19 | 87.41 |
| NSE | 0.741 | 0.041 | 0.660-0.821 | <0.001 | 58.14 | 91.23 | 71.33 |
| NSE+sTIM3 | 0.831 | 0.035 | 0.763-0.899 | <0.001 | 81.40 | 89.47 | 84.62 |
| NSE+sLAG3 | 0.833 | 0.034 | 0.767-0.898 | <0.001 | 89.53 | 73.68 | 83.22 |
| NSE+sTIM3+sLAG3 | 0.863 | 0.030 | 0.804-0.923 | <0.001 | 94.19 | 71.93 | 85.31 |
| CEA+CYFRA211 | 0.920 | 0.023 | 0.875-0.964 | <0.001 | 83.72 | 92.98 | 87.41 |
| CEA+CYFRA211+sTIM3 | 0.936 | 0.020 | 0.898-0.975 | <0.001 | 89.53 | 91.23 | 90.21 |
| CEA+CYFRA211+sLAG3 | 0.950 | 0.017 | 0.917-0.983 | <0.001 | 94.19 | 75.44 | 86.71 |
| CEA+CYFRA211+NSE | 0.932 | 0.021 | 0.891-0.973 | <0.001 | 90.70 | 84.21 | 88.11 |
| CEA+CYFRA211+NSE+sTIM3 | 0.946 | 0.018 | 0.910-0.982 | <0.001 | 94.19 | 82.46 | 89.51 |
| CEA+CYFRA211+NSE+sLAG3 | 0.953 | 0.018 | 0.918-0.987 | <0.001 | 97.67 | 70.18 | 86.71 |
